# Investigating the Molecular Mechanisms of Organophosphate and Pyrethroid Resistance in the Fall Armyworm *Spodoptera frugiperda*

**DOI:** 10.1371/journal.pone.0062268

**Published:** 2013-04-17

**Authors:** Renato A. Carvalho, Celso Omoto, Linda M. Field, Martin S. Williamson, Chris Bass

**Affiliations:** 1 Department of Biological Chemistry and Crop Protection, Rothamsted Research, Harpenden, Hertfordshire, United Kingdom; 2 Department of Entomology and Acarology, University of São Paulo (ESALQ/USP)-Piracicaba, São Paulo, Brazil; University of Crete, Greece

## Abstract

The fall armyworm *Spodoptera frugiperda* is an economically important pest of small grain crops that occurs in all maize growing regions of the Americas. The intensive use of chemical pesticides for its control has led to the selection of resistant populations, however, to date, the molecular mechanisms underlying resistance have not been characterised. In this study the mechanisms involved in the resistance of two *S. frugiperda* strains collected in Brazil to chlorpyrifos (OP strain) or lambda-cyhalothrin (PYR strain) were investigated using molecular and genomic approaches. To examine the possible role of target-site insensitivity the genes encoding the organophosphate (acetylcholinesterase, AChE) and pyrethroid (voltage-gated sodium channel, VGSC) target-site proteins were PCR amplified. Sequencing of the *S. frugiperda ace-1* gene identified several nucleotide changes in the OP strain when compared to a susceptible reference strain (SUS). These result in three amino acid substitutions, A201S, G227A and F290V, that have all been shown previously to confer organophosphate resistance in several other insect species. Sequencing of the gene encoding the VGSC in the PYR strain, identified mutations that result in three amino acid substitutions, T929I, L932F and L1014F, all of which have been shown previously to confer knockdown/super knockdown-type resistance in several arthropod species. To investigate the possible role of metabolic detoxification in the resistant phenotype of the OP and PYR stains all EST sequences available for *S. frugiperda* were used to design a gene-expression microarray. This was then used to compare gene expression in the resistant strains with the susceptible reference strain. Members of several gene families, previously implicated in metabolic resistance in other insects were found to be overexpressed in the resistant strains including glutathione S-transferases, cytochrome P450s and carboxylesterases. Taken together these results provide evidence that both target-site and metabolic mechanisms underlie the resistance of *S. frugiperda* to pyrethroids and organophosphates.

## Introduction

The fall armyworm *Spodoptera frugiperda* (JE Smith) (Lepidoptera: Noctuidae) is a polyphagous species native to tropical regions of the American continent. In Brazil this species is one of the most destructive and economically important insect pests of maize and also causes damage to other crops including soybean, cotton, rice, sorghum and vegetables [1], [2]. The distribution of *S. frugiperda*, although limited to warm climates, covers large geographic areas, largely due to the significant dispersal ability of adults which has allowed it to spread rapidly throughout the range of its host species [3]. The high infestation rate of *S. frugiperda* and the major economic losses it causes has led to a reliance on intensive application of chemical insecticides for control. Unfortunately the widespread and sometimes indiscriminate use of insecticides has contributed to the development of populations with resistance to several different insecticide classes including organophosphates, carbamates, pyrethroids and benzoylureas [4], [5], [6], [7].

The first report of insecticide resistance in *S. frugiperda* was to the carbamate insecticide carbaryl [8]. Since then high levels of resistance have been reported in field populations from North Florida to several pyrethroid and organophosphate insecticides [5], [6]. Resistance has also been observed in laboratory-selected populations which have been described with resistance ratios of more than 40-fold to a given pyrethroid compound [9]. In Brazil, resistance to pyrethroids has also been reported in *S. frugiperda* with a population described with resistance ratios of approximately 13-fold to lambda-cyhalothrin [4].

Biochemical characterization of resistance to pyrethroids and organophosphates in *S. frugiperda* has suggested that both insensitivity of the target site and detoxification of insecticides by metabolic enzymes underlie resistance [7]. Furthermore, a study on the genetics of resistance in *S. frugiperda* to a carbamate (methomyl) and pyrethroid (lambda-cyhalothrin) has indicated that multiple recessive genes are involved [10]. In both studies, however, the specific mutations/genes involved were not identified.

In other insect species resistance to pyrethroids and organophosphates has been most commonly associated with structural alteration (mutation) of the genes encoding target-site proteins (target-site resistance), and/or enhanced expression of metabolic enzymes that break down or sequester the insecticide before it reaches the target (metabolic resistance). For target-site resistance, a relatively small number of highly conserved point mutations have been identified in the genes encoding the voltage-gated sodium channel in insect species with resistance to pyrethroids or in the gene encoding the acetylcholinesterase (AChE) enzyme of species with resistance to organophosphates and carbamates [11], [12]. For metabolic resistance, genomic changes which lead to gene amplification, overexpression and/or modification of genes encoding members of the glutathione S-transferases (GSTs), cytochrome P450s (P450s) and carboxylesterases (CEs) have been most frequently identified in a range of insect species with resistance to pyrethroids, organophosphates and carbamates [13].

Of the 136 products registered for control of *S. frugiperda* in Brazil (as of 2013), 78 were pyrethroids or organophosphates [14] and therefore knowledge of the frequency and distribution of resistance mechanisms to these insecticides is urgently required. Towards this end, in this study we investigated the molecular mechanisms associated with resistance in two strains of *S. frugiperda* collected in Brazil and selected with either chlorpyrifos (OP strain) or lambda-cyhalothrin (PYR strain). The genes encoding the target sites of both insecticide classes were characterized and the frequencies of mutations at known resistance ‘hot spots’ were examined in these populations. In order to identify candidate genes potentially involved in metabolic resistance a microarray was designed based on all available *S. frugiperda* EST sequences and used to identify specific genes that are overexpressed in the resistant strains.

## Materials and Methods

### 1. Insect strains

The *S. frugiperda* strains used in this study were obtained from the Department of Entomology and Acarology, University of São Paulo (ESALQ/USP), Piracicaba, São Paulo, Brazil. The organophosphate (OP) and pyrethroid (PYR) resistant strains were selected from *S. frugiperda* populations collected in cornfields located in Minas Gerais and Mato Grosso States, Brazil, respectively, in 2008. Approximately 200 larvae were obtained from each location (with the permission of the land owner), after reports of control failures with the use of organophosphates or pyrethroid insecticides. The OP strain was maintained under selection (every other generation for 3 years) with chlorpyrifos (at increasing discriminating doses from 100 µg up to 400 µg of insecticide per g of insect in topical bioassays) and the PYR strain with lambda-cyhalothrin (from 8.4 µg up to 27 µg of insecticide per g of insect). The susceptible reference strain (SUS) has been maintained in the laboratory since 1998 without exposure to insecticides. All strains were maintained on artificial diet based on lima bean, wheat germ and yeast [15], at 25±1°C with a 16∶8 L∶D photoperiod as described previously [16].

### 2. Bioassays

Topical bioassays were used to characterize the dose-mortality response of the SUS and resistant (OP and PYR) *S. frugiperda* strains. The insecticides (chlorpyrifos 99.0% and lambda-cyhalothrin 87.4% technical grade) were dissolved in acetone and a 1 µL droplet of different concentrations was dispensed onto the thoracic notum of third instar larvae with an automatic micro-applicator (Burkard Manufacturing, Rickmansworth, England). Controls were treated with acetone alone. After treatment, larvae were transferred individually into a cell of a 24-well plate (Corning) and provided with approximately 1 g of artificial diet. Twenty four larvae per replicate were treated at each insecticide concentration and all tests were replicated four times. Mortality was assessed 24 h after treatment and the larvae were considered dead if they were unable to move in a coordinated manner when disturbed with a needle. Dose-mortality regression and the dose required to kill 50% (LD_50_) were estimated by Probit analysis (LeOra Software). Resistance ratios (RR) were estimated at the LD_50_ level as RR  =  LD_50_ of resistant strains/LD_50_ of the SUS strain.

### 3. Target site amplification

#### 3.1 Acetylcholinesterase

To identify putative alterations in the acetylcholinesterase *ace-1* gene sequence between susceptible and resistant *S. frugiperda* strains, primers were designed in conserved regions of lepidopteran *ace-1* sequences available in GenBank (shown in Table S1). These were then used to amplify approximately 1 kb of the *ace-1* coding region containing the majority of the mutation sites previously reported to confer resistance in a range of other insect species. To estimate mutation frequencies in each strain total RNA was extracted from 20 individuals of the OP and SUS strain using Trizol and following the manufacturer's instructions. Genomic DNA was removed by DNase I digestion using DNA-free DNase treatment and removal reagent (Ambion). The quality and quantity of RNA pools were assessed by spectrophotometry (Nanodrop Technologies) and by running an aliquot on a 1.2% agarose gel. 2 µg of RNA sample was then used for cDNA synthesis using Superscript III and random hexamers (Invitrogen) according to the manufacturer's instructions. A semi-nested PCR approach was employed using AceF2Lep and AceSfR1 primers in a primary P CR reaction (all primers are listed in Table S1) followed by a second round of PCR using a different reverse primer, AceSfR2. PCR reactions (20 µL) consisted of 10 µL of DreamTaq Green ™ 2X PCR Master Mix (Fermentas), 6 µL distilled water, 1 µL of each primer (10 mM) and 2 µL of cDNA. Temperature cycling conditions were 95°C for 1 min followed by 35 cycles of 95°C for 30 seconds, 55°C for 30 seconds and 72°C for 2 minutes, followed by a final extension of 72°C for 10 minutes. Amplified fragments were visualized on 1% agarose gels, purified using the Wizard® SV Gel kit and PCR Clean-Up System (Promega) and ‘direct sequenced’ using the same primers used in the last round of PCR. Sequences were analyzed using the program Vector NTI® (Invitrogen). A consensus sequence was used for amino acid sequence prediction and to perform alignments with other athropod species.

#### 3.2 Voltage gated sodium channel

To identify putative alterations in the VGSC gene sequence between susceptible and resistant *S. frugiperda* strains, degenerate primers were designed based on all the lepidopteran VGSC gene sequences available in GenBank (Table S1). These were used to amplify approximately 350 bp of the IIS4-IIS6 region of the VGSC encompassing the five major mutation sites associated with pyrethroid resistance in other arthropod species. RNA extraction and PCR amplification conditions were as above but used primers NaChF1Lep and NaChR1Lep in the primary PCR and NaChF1Lep and NaChR2Lep in the second round of PCR (primer sequences are shown in Table S1), using 58°C as the annealing temperature. Based on the sequences obtained specific primers were designed and used to genotype 20 individuals of the SUS strain and 14 individuals from the PYR strain using primers NaChF1Sf and NAChR1Sf in primary PCR and NaChF2Sf and NaChR1Sf in a second round of PCR. PCR products were analysed, purified and sequenced as described above.

### 4. Microarray procedures

A SurePrint HD (8×15 k) expression array was designed using the Agilent eArray platform. The base composition and the best probe methodologies were selected to design sense orientation 60-mer probes with a 3′ bias. The *S. frugiperda* EST database (SPODOBASE) was used as the reference transcriptome [17]. These sequences are derived from 8 cDNA libraries: Sf1F from fat body, Sf1H from hemocytes, Sf1M from midgut, Sf1P from pools of various tissues, Sf2H from immune challenged hemocytes, Sf2L from Sf21 cell lines, Sf2M from xenobiotic induced midguts and Sf9L from Sf9 cell lines. All assembled contigs and singlets were provided by the website maintainers. The BLAST2GO software v.2.3.1 (http://www.blast2go.org) was used to annotate the EST database, as described in [18]. 60-mer probes were designed for all 7,552 assembled contigs and 5,519 annotated singlets (BlastX), totaling 13,071 sequences. For contigs encoding detoxification enzymes (P450s, GSTs and CEs) three probes were designed. Additional probe groups for 15 control genes were also included. This microarray was used to compare gene expression in each resistant strain (OP and PYR) with the SUS strain. Total RNA was extracted from four pools of 5 second instar larvae, using the Isolate RNA Mini Kit (Bioline) according to the manufacturer's protocol. 200 ng of each total RNA was used to generate labelled cRNA, which was hybridized to arrays and washed as described in Agilent's Quick Amp Labelling Protocol (Version 6.5). The microarray experiment consisted of four biological replicates and incorporated a dye swap design whereby the Cy3 and Cy5 labels were swapped between resistant and susceptible strains. Microarrays were scanned with an Agilent G2505C US10020348 scanner, and fluorescent intensities of individual spots were obtained using the Agilent Feature Extraction software with default Agilent parameters. Data normalization, filtering, dye flipping and statistical analysis were performed using the GeneSpring GX 11 suite (Agilent). For statistical analysis, a t-test against zero using the Benjamini-Hochberg false discovery rate (FDR) method for multiple testing corrections was used to detect significantly differentially expressed genes. Genes meeting a p value cut-off of 0.01 and showing a transcription ratio >2-fold in either direction were considered to be differentially transcribed between the two strains. All microarray data were MIAME compliant and were submitted to the Gene Expression Omnibus (GEO) database under accession number GSE43295.

### 5. Quantitative RT-PCR

Quantitative RT-PCR was used to validate microarray data by examining the expression profile of ∼10 genes for each resistant vs. susceptible comparison. Primers were designed to amplify a fragment of 90–150 bp in size and are listed in table S1. Total RNA was prepared as described earlier and four micrograms was used for cDNA synthesis using Superscript III and random hexamers (Invitrogen) according to the manufacturer's instructions. PCR reactions (20 µL) contained 4 µL of cDNA (10 ng), 10 µL of SensiMix SYBR Kit (Bioline), and 0.25 mM of each primer. Samples were run on a Rotor-Gene 6000 (Corbett Research) using the temperature cycling conditions of: 10 minutes at 95°C followed by 40 cycles of 95°C for 15 s, 57°C for 15 s and 72°C for 20 s. A final melt-curve step was included post-PCR (ramping from 72°C–95°C by 1°C every 5 s) to confirm the absence of any non-specific amplification. The efficiency of PCR for each primer pair was assessed using a serial dilution of 100 ng to 0.01 ng of cDNA. Each qRT-PCR experiment consisted of three independent biological replicates with two technical replicates for each. Data were analysed according to the ΔΔCT method [19], using the geometric mean of two selected housekeeping genes (28S which encodes a ribosomal subunit, and *EF* which encodes elongation factor) for normalization according to the strategy described previously [20]. The standard deviation and 95% confidence limits of 2-ΔΔCt were determined from the triplicate samples. Significance between strains was assumed if the 95% confidence limits of the 2-ΔΔCt values did not overlap.

## Results

### 1. Bioassays

In topical bioassays the *S. frugiperda* OP and PYR strains showed approximately 18- and 28-fold resistance to chlorpyrifos and lambda-cyhalothrin respectively compared to the SUS strain (Table 1).

**Table 1:** Dose-mortality response of *S. frugiperda* strains to lambda-cyhalothrin and chlorpyrifos.

Insecticide	Strain	n^a^	Slope(±SE)	LD_50_ b(95% CI)	RR^c^(95% CI)
Chlorpyrifos	SUS	611	1.31(±0.10)	19.78(17.48–22.15)	-
	OP	588	2.81(±0.211)	357.03(263.77–486.99)	18.1(15.4–21.1)
Lambda-cyhalothrin	SUS	720	1.63(±0.108)	0.30(0.20–0.42)	-
	PYR	624	3.11(±0.215)	8.47(6.72–10.52)	28.2(23.2–34.4)

a number of larvae tested.

b µg of insecticide/g of insect.

c LD_50_ of resistant strains/LD_50_ of the SUS strain.

### 2. Acetylcholinesterase

**Figure 1:**
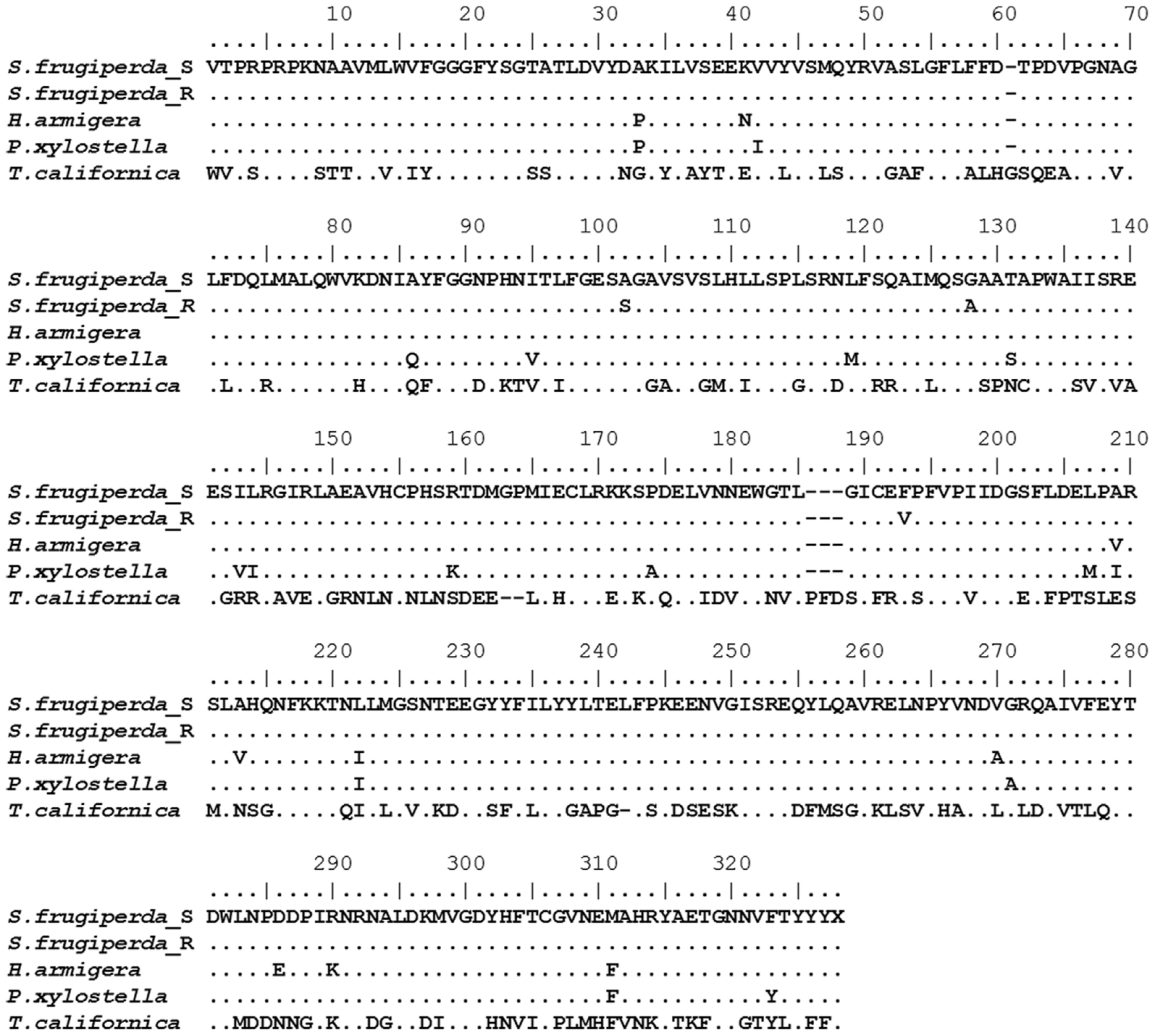
Alignment of the predicted amino acid sequence of the *ace-1* gene amplified from resistant (*S. frugiperda*_R) and susceptible (*S. frugiperda*_S) *S. frugiperda* strains with *ace-1* cDNA sequences from *H. armigera* (DQ064790.1), *P. xylostella* (AY773014.2) and *T. californica* (GI|64389). The positions of the mutations A201S, G227A, F290V are highlighted.

Using primers based on lepidopteran *ace-1* sequences available in GenBank a 972 bp fragment of the *S. frugiperda ace-1* gene was RT-PCR amplified, cloned and sequenced (Genbank accession numbers KC435023 and KC435024). This fragment encodes 324 amino acids and encompasses the majority of mutation sites previously associated with organophosphate resistance in other arthropod species [12]. The obtained sequence shows highest similarity to the orthologous gene from other Lepidoptera such as *Helicoverpa armigera* and *Plutella xylostella* (Figure 1).

Three substitutions were found in the predicted amino acid sequence of the OP strain when compared to that of the SUS strain: A201S, G227A and F290V (numbering corresponding to *Torpedo californica* mature enzyme). To estimate the frequency of AChE mutations in the OP strain 20 individuals were genotyped by direct sequencing. The A201S allele was present at relatively low frequency (17.5%) while G227A and F290V were present at higher frequency (67.5% and 32.5% respectively). The G227A and F290V mutations were commonly observed in the same individual in the heterozygous state but were never found together in a single insect in the homozygous form. The A201S mutation was only found in a single insect in the homozygous form where it was observed with the G227A mutation (Table 2). No individuals of the SUS strain had any of these mutations.

**Table 2:** Genotype of *ace-1* mutations in the OP (organophosphate resistant) strain.

	Genotype		
Strain/Individual	AA201	AA227	AA290
SUS 1–20	Ala	Gly	Phe
OP 1	Ala/Ser	Gly/Ala	Val
OP 2	Ala	Gly/Ala	Phe/Val
OP 3	Ala/Ser	Ala	Phe
OP 4	Ala	Gly/Ala	Phe/Val
OP 5	Ala	Ala	Phe
OP 6	Ala/Ser	Ala	Phe
OP 7	Ala	Gly/Ala	Phe/Val
OP 8	Ala	Gly/Ala	Phe/Val
OP 9	Ala	Gly/Ala	Phe/Val
OP 10	Ala	Gly	Val
OP 11	Ser	Ala	Phe
OP 12	Ala	Gly/Ala	Phe/Val
OP 13	Ala	Ala	Phe
OP 14	Ala	Ala	Phe
OP 15	Ala	Ala	Phe
OP 16	Ala/Ser	Gly/Ala	Phe/Val
OP 17	Ala	Gly/Ala	Phe/Val
OP 18	Ala	Ala	Phe
OP 19	Ala/Ser	Ala	Phe
OP 20	Ala	Gly	Val

For reference the genotype of the SUS (susceptible reference) strain is included in the first row.

### 3. Voltage gated sodium channel

**Figure 2:**
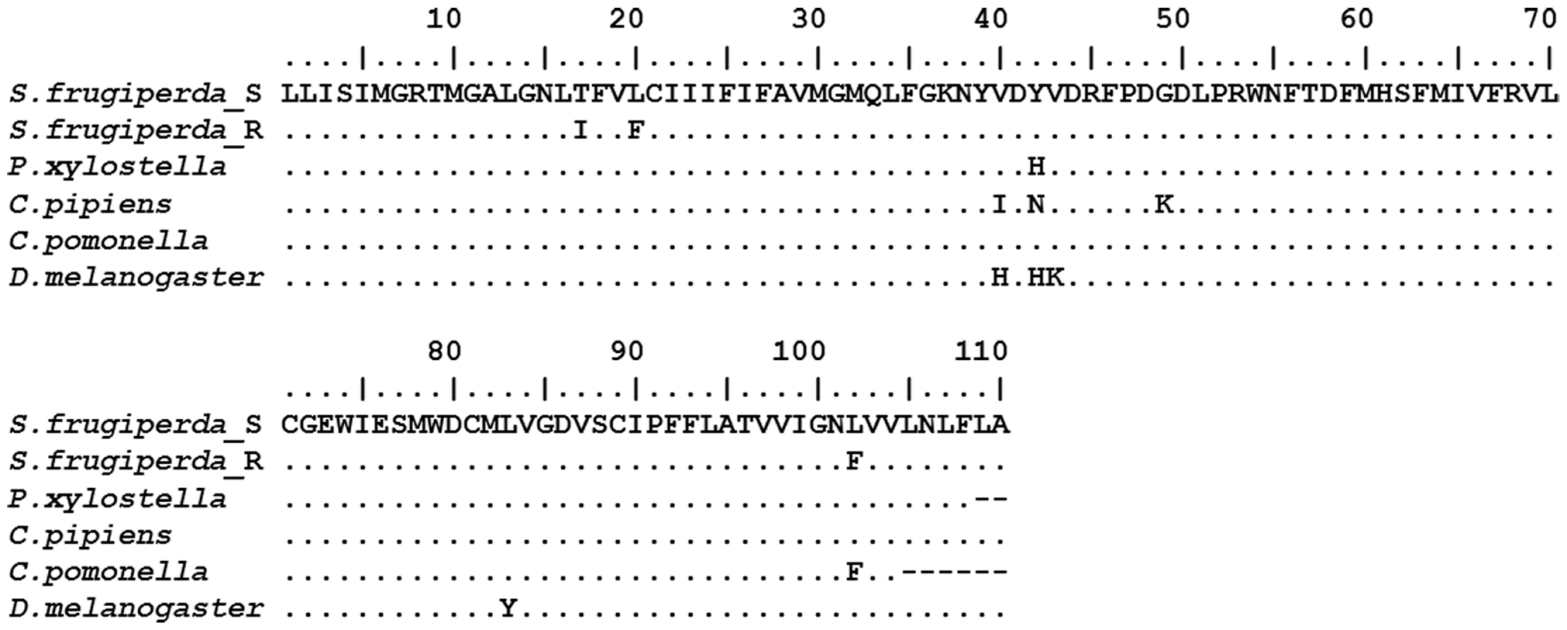
Alignment of the predicted amino acid sequence of a partial cDNA fragment encoding the voltage gated sodium channel amplified from resistant (*S. frugiperda*_R) and susceptible (*S. frugiperda*_S) *S. frugiperda* strains with other species such as *Drosophila melanogaster* (gi|1110475), *Plutella xylostella* (gi|2769535), *Culex pipiens* (gi|89213629) and *Cydia pomonella* (gi|53988535). The position of mutations T929I, L932F and L1014F found in *S. frugiperda* are highlighted.

Using primers based on lepidopteran sequences available in GenBank a 330 bp fragment of the gene encoding the IIS4-IIS6 region of the *S. frugiperda para*-type VGSC was amplified by RT-PCR, cloned and sequenced (Genbank accession numbers KC435025 and KC435026). This fragment encodes 110 amino acids and encompasses the five major mutation sites previously reported to be implicated in conferring kdr-type resistance to pyrethroids across a range of different insects [11]. The predicted amino acid sequence of the obtained fragment shows high similarity to the orthologous gene in several other insects including *Plutella xylostella*, *Culex pipiens, Cydia pomonella*, and *Drosophila melanogaster* (Figure 2). Three substitutions were found in the predicted amino acid sequence of the PYR strain when compared to the SUS strain: T929I, L932F and L1014F. In contrast to what we found for the AChE mutations, the frequency of VGSC mutant alleles was very low in the PYR strain, with only one individual containing both T929I and L1014F substitutions and another single individual with the L932F substitution.

### 4. Microarray analysis

#### 4.1 Chlorpyrifos resistant strain

Microarray analysis identified 497 probes as significantly differentially transcribed (more than 2-fold over or under expressed, p<0.01) between the OP strain and the susceptible SUS strain (Table S2). 315 probes had elevated expression in the OP strain and of these, 120 had been previously annotated using the program Blast2Go. The top 40 annotated over expressed probes/ESTs are shown in Table 3 and several of these may be considered potential candidates for causing insecticide resistance. These included probes corresponding to ESTs encoding GSTs (10), P450s (10) and CEs (2), enzymes that have been implicated in insecticide resistance in many arthropod species [13]. Two sequences encoding CEs were identified as over-expressed in the OP strain. The level of expression of these sequences was very high, particularly for the EST (Sf1P09555-5-1) with high sequence similarity to E4 carboxylesterase (21-fold), an enzyme that has been shown previously to confer organophosphate resistance. In addition, several other ESTs were found with high levels of expression that encode enzymes that may be capable of metabolizing xenobiotics including short chain dehydrogenases (Sf1M06421-3-1), aldehyde dehydroxygenases (Sf1F06267-5-1) and glucosyl-glucuronosyl transferases (Sf2M05474-5-1).

**Table 3:** The top 40 annotated probes/ESTs over expressed by microarray in the *S. frugiperda* OP strain.

ProbeName	p-value	Fold change	PrimaryAccession	Description (blastx)
CUST_7605_PI426916786	0,0032	125,87	Sf1F00413-3-1	kda hemolymph protein
CUST_8476_PI426916786	0,0084	37,00	Sf1H01750-3-1	c1a cysteine protease precursor
CUST_315_PI426916783	0,0084	26,65	Sf2M01967-5-1-Contig1	microsomal glutathione s-transferase 1
CUST_440_PI426916783	0,0054	21,09	Sf1M01746-3-1	glutathione s-transferase sigma
CUST_67_PI426916783	0,0010	21,05	Sf1P09555-5-1-Contig1	esterase fe4
CUST_197_PI426916783	0,0063	17,31	Sf1P01950-5-1-Contig3	pyruvate dehydrogenase
CUST_8635_PI426916786	0,0099	16,78	Sf1H07691-3-1	cecropin b
CUST_9791_PI426916786	0,0026	16,20	Sf2M05474-5-1	glucosyl glucuronosyl transferases
CUST_7591_PI426916786	0,0009	16,10	Sf2H04339-5-1	26s protease regulatory subunit 7
CUST_11088_PI426916786	0,0045	15,71	Sf1F00509-5-1	p27k_galme ame: full = 27 kda hemolymph protein
CUST_8033_PI426916786	0,0045	15,18	Sf2M07369-3-1	alkaline nuclease
CUST_184_PI426916783	0,0095	15,12	Sf1M10453-3-1-Contig1	glutathione s-transferase
CUST_11087_PI426916786	0,0021	14,89	Sf1F10140-5-1	p27k_galme ame: full = 27 kda hemolymph protein
CUST_508_PI426916783	0,0089	14,75	Sf1P04772-5-1-Contig1	cytochrome p450
CUST_499_PI426916783	0,0048	14,21	Sf1P14935-5-1-Contig1	cytochrome p450
CUST_195_PI426916783	0,0063	14,18	Sf1P01950-5-1-Contig2	pyruvate dehydrogenase
CUST_227_PI426916783	0,0060	13,62	Sf2L01018-5-1-Contig1	glutathione s-transferase
CUST_190_PI426916783	0,0071	12,36	Sf1P01950-5-1-Contig1	glutathione s-transferase
CUST_10422_PI426916786	0,0097	12,29	Sf2M14080-3-1	juvenile hormone epoxide hydrolase
CUST_226_PI426916783	0,0019	11,79	Sf2L01018-5-1-Contig1	glutathione s-transferase
CUST_209_PI426916783	0,0036	11,50	Sf2M00801-5-1-Contig1	glutathione s-transferase
CUST_11493_PI426916786	0,0014	11,13	Sf1P15441-5-1	protein transport protein sec23
CUST_194_PI426916783	0,0049	11,05	Sf1P01950-5-1-Contig2	pyruvate dehydrogenase
CUST_10201_PI426916786	0,0034	10,39	Sf1P09780-5-1	imp dehydrogenase gmp reductase
CUST_11972_PI426916786	0,0054	10,08	Sf1M06421-3-1	short-chain dehydrogenase
CUST_8014_PI426916786	0,0022	9,91	Sf1F06267-5-1	aldehyde dehydroxygenase
CUST_335_PI426916783	0,0061	9,42	Sf1F10827-3-1	glutathione s-transferase
CUST_11192_PI426916786	0,0001	9,25	SF9L00826	phd finger-like domain-containing protein 5a
CUST_8435_PI426916786	0,0009	9,18	Sf1P20209-5-1	bis(5 -nucleosyl)-tetraphosphatase
CUST_198_PI426916783	0,0084	8,93	Sf1P01950-5-1-Contig3	pyruvate dehydrogenase
CUST_677_PI426916783	0,0011	8,65	Sf2M09131-5-1	cytochrome p450
CUST_14_PI426916783	0,0049	8,59	Sf2M00974-5-1-Contig1	carboxyl choline esterase cce016a
CUST_9640_PI426916786	0,0024	8,28	Sf1F07895-3-1	fatty acid binding protein
CUST_176_PI426916783	0,0017	7,97	Sf1F00968-3-1-Contig4	glutathione s-transferase
CUST_10462_PI426916786	0,0033	7,91	Sf1F01201-3-1	l-xylulose reductase
CUST_228_PI426916783	0,0080	7,81	Sf2L01018-5-1-Contig1	glutathione s-transferase
CUST_191_PI426916783	0,0071	7,76	Sf1P01950-5-1-Contig1	pyruvate dehydrogenase
CUST_11211_PI426916786	0,0001	7,51	Sf2H08686-3-1	phosphatidylinositol-glycan biosynthesis class f pro
CUST_8265_PI426916786	0,0094	7,44	Sf1P14042-5-1	n -(beta-n-acetylglucosaminyl)-l-asparaginase
CUST_10187_PI426916786	0,0028	7,02	SF9L03509	immediate early response 3-interacting protein 1

#### 4.2 Lambda-cyhalothrin resistant strain

Microarray analysis identified 535 probes as significantly differentially transcribed (more than 2-fold over-/under-expressed, p<0.01) between the PYR-selected strain and the susceptible standard SUS (Table S3). 238 probes had elevated expression in the PYR strain and of these 92 had been previously annotated (BlastX). The top 40 annotated over expressed probes/ESTs are shown in Table 4. For genes encoding enzymes involved in metabolic detoxification, GST genes were by far the most abundant overexpressed gene family with 27 probes representing 10 ESTs. By contrast, only single ESTs encoding a P450 (Sf2H09360-3-1) and a carboxylesterase (Sf1P26308-5-1) were found to be overexpressed. Other ESTs overexpressed in the PYR strain associated with xenobiotic metabolism included two UDP-glucosyltransferases (Sf2M12870-3-1 and Sf2H08497-3-1) and a carbonyl reductase (Sf1P07238-5-1). When overexpressed EST lists are compared between the OP and PYR strains only six probes are overexpressed in both resistant strains (representing five ESTs, encoding a hymolymph protein (Sf1F00509-5-1), a pyruvate dehydrogenase (Sf1P01950-5) and three GSTs (Sf2L01018-5-1, Sf1F10827-3-1 and Sf1F00968-3-1).

**Table 4:** The top 40 annotated probes/ESTs over expressed by microarray in the *S. frugiperda* PYR strain.

ProbeName	p-value	Fold change	PrimaryAccession	Blast2go description (tblastx)
CUST_10566_PI426916786	0,0009	26,08	Sf1F01577-3-1	lysozyme
CUST_197_PI426916783	0,0046	23,87	Sf1P01950-5-1-Contig3	pyruvate dehydrogenase
CUST_10570_PI426916786	0,0014	18,18	Sf1F01577-3-1	lysozyme
CUST_9158_PI426916786	0,0017	17,35	Sf1F07575-3-1	cytochrome oxidase subunit i
CUST_226_PI426916783	0,0002	16,88	Sf2L01018-5-1-Contig1	glutathione s-transferase
CUST_170_PI426916783	0,0001	14,68	Sf1F00968-3-1-Contig2	glutathione s-transferase
CUST_167_PI426916783	0,0013	14,60	Sf1F00968-3-1-Contig1	glutathione s-transferase
CUST_194_PI426916783	0,0003	14,52	Sf1P01950-5-1-Contig2	pyruvate dehydrogenase
CUST_169_PI426916783	0,0008	14,40	Sf1F00968-3-1-Contig2	glutathione s-transferase
CUST_198_PI426916783	0,0004	13,57	Sf1P01950-5-1-Contig3	pyruvate dehydrogenase
CUST_10557_PI426916786	0,0019	13,51	Sf1H02510-3-1	lysozyme
CUST_209_PI426916783	0,0020	13,06	Sf2M00801-5-1-Contig1	glutathione s-transferase
CUST_227_PI426916783	0,0001	12,62	Sf2L01018-5-1-Contig1	glutathione s-transferase
CUST_336_PI426916783	0,0017	12,25	Sf1F10827-3-1	glutathione s-transferase
CUST_195_PI426916783	0,0004	12,22	Sf1P01950-5-1-Contig2	pyruvate dehydrogenase
CUST_12816_PI426916786	0,0028	12,16	Sf2M12870-3-1	uridine diphosphate glucosyltransferase
CUST_228_PI426916783	0,0009	11,29	Sf2L01018-5-1-Contig1	glutathione s-transferase
CUST_190_PI426916783	0,0013	11,20	Sf1P01950-5-1-Contig1	pyruvate dehydrogenase
CUST_9656_PI426916786	0,0083	10,42	Sf1P26318-5-1	ferric-chelate reductase 1homolog isoform 1
CUST_12703_PI426916786	0,0035	10,33	Sf2H08497-3-1	udp-glucosyltransferase
CUST_192_PI426916783	0,0059	10,16	Sf1P01950-5-1-Contig1	pyruvate dehydrogenase
CUST_11088_PI426916786	0,0058	9,67	Sf1F00509-5-1	p27k_galme ame: full = 27 kda hemolymph pro
CUST_10187_PI426916786	0,0012	9,18	SF9L03509	immediate early response 3-interacting protei
CUST_8948_PI426916786	0,0006	9,04	Sf2L01305-5-1	copia-like retrotransposable element
CUST_176_PI426916783	0,0089	8,33	Sf1F00968-3-1-Contig4	glutathione s-transferase
CUST_196_PI426916783	0,0004	8,21	Sf1P01950-5-1-Contig3	pyruvate dehydrogenase
CUST_11701_PI426916786	0,0002	7,90	Sf1F01613-3-1	ribosomal protein s11 isoform 1
CUST_191_PI426916783	0,0001	7,76	Sf1P01950-5-1-Contig1	pyruvate dehydrogenase
CUST_11211_PI426916786	0,0053	7,60	Sf2H08686-3-1	phosphatidylinositol-glycan biosynthesis class f
CUST_175_PI426916783	0,0054	7,38	Sf1F00968-3-1-Contig4	glutathione s-transferase
CUST_8765_PI426916786	0,0007	7,17	Sf1P12294-5-1	checkpoint protein
CUST_335_PI426916783	0,0001	6,88	Sf1F10827-3-1	glutathione s-transferase
CUST_11096_PI426916786	0,0064	6,72	Sf2H09127-3-1	palmitoyltransferase zdhhc2
CUST_11456_PI426916786	0,0090	6,43	Sf1P21758-5-1	protein ltv1 homolog
CUST_10563_PI426916786	0,0057	6,26	Sf1F02768-3-1	Lysozyme
CUST_8528_PI426916786	0,0005	6,07	Sf1P07238-5-1	carbonyl reductase
CUST_10266_PI426916786	0,0040	5,73	Sf1P19974-5-1	intraflagellar transport protein 140 homolog
CUST_8265_PI426916786	0,0094	5,66	Sf1P14042-5-1	n -(beta-n-acetylglucosaminyl)-l-asparaginase
CUST_11881_PI426916786	0,0042	5,58	Sf1M05505-5-1	serine protease 31
CUST_9307_PI426916786	0,0070	5,53	Sf1P23771-5-1	delta –desaturase

### 5. qRT-PCR

**Figure 3:**
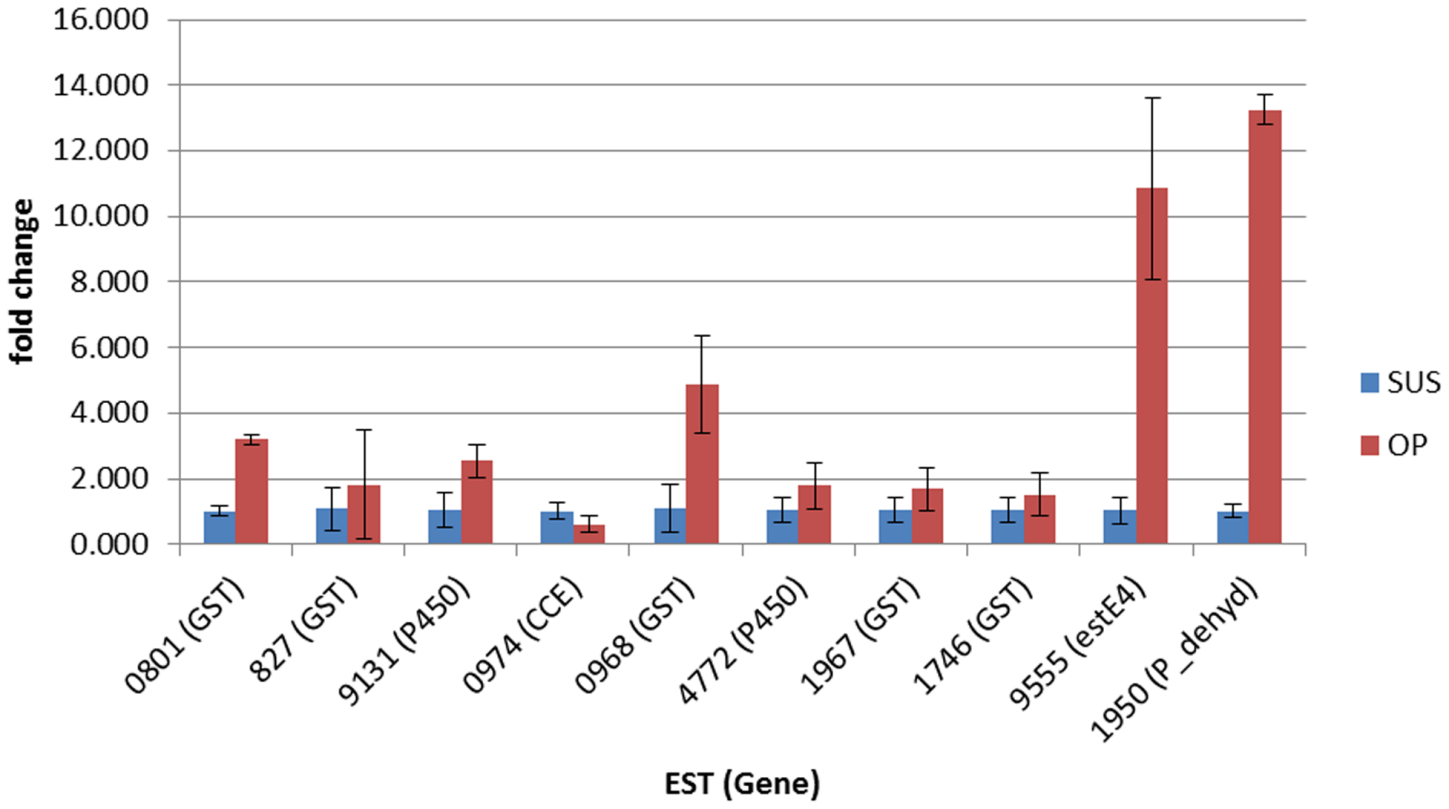
Fold change in expression of selected ESTs between the SUS (blue columns) and OP strain (red columns) in qPCR analysis. Error bars display 95% confidence intervals.

**Figure 4:**
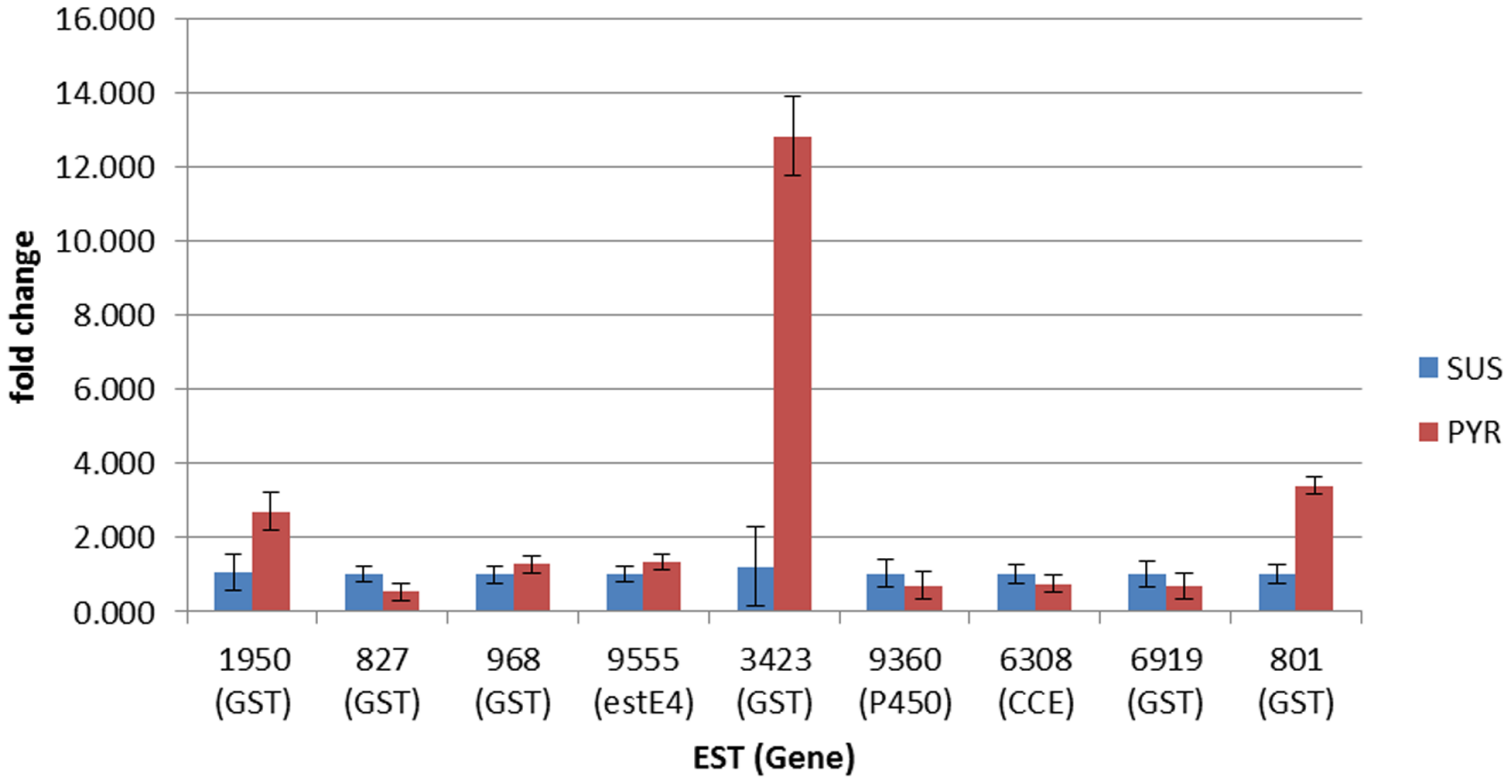
Fold change in expression of selected ESTs between SUS (blue columns) and PYR strain (red columns) in qPCR analysis. Error bars display 95% confidence intervals.

Real-time quantitative PCR (qPCR) was used to validate the microarray results and identify genes most likely to be involved in resistance by examining the expression profile of ∼10 selected genes for each array comparison (see Figures 3 and 4). For the SUS vs OP comparison a significant difference in gene expression between the two strains was confirmed for five out of ten ESTs (Figure 3). For the SUS vs PYR comparison a significant difference in gene expression between the two strains was confirmed for three out of nine ESTs with the expression of a further EST (contig 9555), agreeing with array data as not showing significant differences in expression between the two strains (Figure 4). Discrepancies in the data obtained from array experiments using the Agilent array platform and qPCR have been reported previously and our results again highlight the importance of qPCR validation of array data. As shown in figure 3 the two most overexpressed genes in qPCR analysis for the OP strain are EST 9555 encoding a carboxylesterase E4-like protein (overexpressed ∼11-fold) and EST 1950 encoding a pyruvate dehydrogenase, overexpressed ∼13-fold). The three ESTs significantly overexpressed in the PYR strain (1950, 3424 and 0801) are all GSTs (figure 4).

## Discussion

Previous studies investigating the biochemical characteristics of resistance to pyrethroids, organophosphates and carbamates in *S. frugiperda* have provided strong evidence that multiple mechanisms underlie resistance, including detoxification by microsomal oxidases, glutathione S-transferases, hydrolases and reductases, and target site insensitivity such as insensitive AChE [5], [6], [7]. In this study our aim was to build on this work but use molecular and genomic approaches to identify the specific mutations involved in target-site resistance and the candidate detoxification enzymes in metabolic resistance.

In our topical bioassays the PYR strain showed moderate levels of resistance (30-fold) to the pyrethroid lambda-cyhalothrin compared to the SUS strain. To investigate whether resistance was mediated by mutation of the pyrethroid target site we cloned and sequenced the IIS4-IIS6 region of the para-type sodium channel which contains many of the mutation sites previously shown to cause kdr-type resistance in a range of different insects. This revealed three kdr/super kdr-type mutations within the PYR strain at known resistance ‘hot-spots’ within this gene region, T929I, L932F and L1014F. The L1014F mutation, first identified in pyrethroid resistant house fly strains, is the most commonly reported kdr-type mutation in a range of arthropod species where it typically confers between 10–30 fold resistance to pyrethroids [21]. Functional characterisation of this mutation in insect channels injected into *Xenopus laevis* oocytes has demonstrated that it confers up to a 17-fold reduction in sensitivity to certain pyrethroids [22]. T929I is a super kdr-type mutation first identified in pyrethroid resistant diamondback moth, *P. xylostella*, which has since been reported in human head lice, *Pediculosis capitis*, maize weevil, *Sitophilus zeamais* and tomato leafminer, *Tuta absoluta* [23], [24], [25], [26]. Functional expression studies in oocytes have demonstrated that T929I in combination with L1014F makes insect sodium channels highly insensitive to both type I and type II pyrethroids and also to DDT [27], [28]. To date, the L932F mutation has only been identified in human head lice, where it is frequently associated with the T929I mutation, and the peach potato aphid *Myzus persicae* where it was associated with L1014F [24], [29]. Heterologous expression of the L932F mutation in oocytes has shown it also reduces channel sensitivity to permethrin [30]. Sequencing individuals of the PYR strain showed all three mutations are at low frequency in this strain with one individual identified that carried both the T929I and L1014F substitutions and another single individual that carried the L932F substitution. At this frequency the three mutations are unlikely to fully explain the resistance phenotype observed in the PYR strain and it is surprising that the maintenance of this strain under selection with lambda cyhalothrin has not selected for a higher frequency of one or more of the three mutations. It is possible that this could be explained by a fitness cost associated with one or more of the mutations. In this regard it is noteworthy that Yoon et al have shown that T929I reduces the expression rate of mutant sodium channels in oocytes suggesting it may carry a fitness cost by impairing sodium channel function [30]. Alternatively, the insecticide dose used for selection may have exerted insufficient selection pressure to preferentially select individuals carrying the mutations.

The second resistant *S. frugiperda* strain investigated in this study, the OP strain, showed moderate resistance (20-fold) to the organophosphate chlorpyrifos compared to the SUS strain in topical bioassays. Cloning of a significant fragment of the *ace-1* gene encoding the target protein of the organophosphate and carbamate insecticides revealed the presence of amino acid substitutions, A201S, G227A and F290V, at three positions implicated previously in OP resistance in several different insect species. Of the three, the A201S mutation was observed at the lowest frequency (17.5%) in the OP strain. This mutation was first reported in the cotton aphid, *Aphis gossypii* where it is associated with insensitivity to a wide range of carbamates and organophosphates [31]. It has subsequently been described in organophosphate resistant strains of the rice stem borer, *Chilo suppressalis*, the oriental fruit fly, *Bactrocera dorsalis* and the diamondback moth, *P. xylostella* [32], [33], [34]. In *P. xylostella* functional expression of susceptible and resistant versions (with A201S in combination with G227A) of ace-1 demonstrated that the resistant version of the protein is less sensitive to the organophosphate paraoxon [34].

The G227A and F290V mutations were observed in the OP strain at higher frequency (67.5% and 32.5% respectively) than A201S. In addition to *P. xylostella* the G227A mutation has also been described in several other insect and mite species [12] and recombinant ace-1 proteins with this substitution have been functionally expressed, showing that this mutation, on its own, confers relatively modest levels of resistance to most organophosphates. F290V has previously been described in the codling moth, *Cydia pomonella*, however, other substitutions of the F290 residue such as F290Y have been described in other insects including *Drosophila* and *M. domestica* [35], [36], [37]. Functional expression of recombinant ace-1 with this mutation has demonstrated that it confers modest levels of resistance [35]. In many insect species where several mutations are observed in the *ace* gene the effects of mutation combinations are additive. For example in *M. domestica*, although the G227A and F290Y each confer a low level of insensitivity, when combined their effect is significantly enhanced [35]. In the *S. frugiperda* OP strain the three AChE mutations were most commonly found in combination in the same individual and likely also act in concert to enhance resistance. Interestingly G227A and F290V were never observed together in the homozygous form in the same individual suggesting that they are not on the same allele in the OP strain. This is in contrast to the A201S mutation which seems to have arisen in a G227A genetic background as it was only observed in combination with this mutation.

To investigate if metabolic detoxification also plays a role in the resistance of the OP and PYR strains we designed a microarray based on all available *S. frugiperda* EST sequences and used it to compare gene expression in the resistant strains with a susceptible reference strain. A number of ESTs that can be considered potential candidates for a role in insecticide resistance, were shown to be overexpressed in the resistant strains. These included ESTs encoding GSTs, P450s and CEs, enzymes that have been implicated in metabolic resistance in many arthropod species. Several other ESTs that encode enzymes capable of metabolizing xenobiotics (and potentially insecticides) including, short chain dehydrogenases, aldehyde dehydroxygenases and glucosyl-glucuronosyl transferases were also overexpressed. In the case of the OP strain of particular relevance was a sequence encoding a CE that was identified as being significantly over-expressed in both microarray (21-fold) and qPCR (∼11-fold) experiments. Overexpression of E4 esterase was first described in *M. persicae* where it confers broad-spectrum resistance to organophosphates and carbamates as a consequence of both sequestration and ester hydrolysis [38]. Purification of this esterase and further functional analysis is therefore warranted. Several ESTs encoding P450s were also overexpressed in the OP strain in microarray analyses and one of these (Sf2M09131-5-1) was confirmed as overexpressed by qPCR ∼3-fold. This EST encodes a P450 with highest sequence similarity to *Spodoptera littoralis* CYP6B50. Members of the P450 CYP6 family have been shown to confer resistance to organophosphates in several insects previously [13]. Further suggestion that this P450 may be involved in resistance is that the EST was derived from a xenobiotic induced midgut *S. frugiperda* library. Several ESTs encoding GSTs were significantly overexpressed in the OP strain in microarray analysis. Q-PCR revealed that two of these were overexpressed (∼3-5-fold) and belong to the epsilon (Sf2m00801-5-1) and sigma GST families (Sf1F00968-3-1). Overexpression of GSTs has previously been associated with resistance to organophosphates in Lepidoptera [39]. In *P. xylostella* GST3 is overproduced in resistant strains, and heterologous expression of PxGSTE1 showed it is capable of metabolising the organophosphate insecticides parathion and methylparathion [39]. Interestingly GST3 also belongs to the epsilon GST family sharing ∼50% sequence identity with the Sf2m00801-5-1 sequence. It is also interesting that Sf2m00801-5-1 is derived from the xenobiotic induced midgut *S. frugiperda* library. In addition to the overexpressed genes that belong to gene families known to be involved in resistance, several other genes were overexpressed in the OP strain that may be capable of metabolizing xenobiotics, including short chain dehydrogenases, aldehyde dehydroxygenases and glucosyl-glucuronosyl transferases. Of these the EST encoding a glucosyl-glucuronosyl transferase (Sf2M05474-5-1) should be prioritized for further future investigation as it was one of the most highly overexpressed genes in microarray analysis of the OP strain, is derived from a xenobiotic induced midgut library, and this family are responsible for the most important detoxification pathway of Phase II drug metabolism in many vertebrates including humans [40].

For the PYR strain, of genes previously implicated in insecticide resistance, those encoding GSTs were by far the most abundant overexpressed gene family. As for the OP strain the EST Sf2m00801-5-1 encoding a GST of the epsilon family was overexpressed in both microarray and qPCR experiments. However, qPCR analysis revealed that another EST (Sf1F03423-5-1), encoding a GST belonging to the sigma family, was overexpressed at a much higher level (∼13-fold in qPCR analysis). Elevated expression of GSTs have been associated previously with pyrethroid resistance in Lepidoptera (*Spodoptera littoralis*) [41]. Furthermore, induction of GSTs by pyrethroid exposure has also been previously reported for *S. frugiperda* [42]. However, in contrast to organophosphates, pyrethroids have not been shown to be directly metabolized by GSTs. Rather studies on pyrethroid-resistant brown planthoppers, *Nilaparvata lugens* with elevated GST activity has suggested they may protects against lipid peroxidation products and oxidative stress that are induced by pyrethroid exposure [43]. In addition, insect GSTs may act by sequestering pyrethroids until they are metabolized by other detoxification enzymes [44]. In this regard ESTs encoding a P450 and two carboxylesterases were upregulated in the PYR strain in microarray experiments, however, qPCR validation showed that the levels of expression of these ESTs is not significantly different in the SUS and PYR strains. Other ESTs associated with xenobiotic metabolism that were overexpressed in the PYR strain in microarray analysis include two UDP-glucosyltransferases and a carbonyl reductase, both of which warrant further investigation.

In summary this study has identified mutations in the genes encoding the VGSC and AChE enzyme of *S. frugiperda* that have been shown previously to confer resistance to pyrethroids and organophosphates respectively in a range of arthropods. However, our analyses have provided further support that resistance to organophosphates and pyrethroids in *S. frugiperda* is multigenic and we have identified a promising list of candidate genes that may also play a role in resistance. The consistency of overexpression of these genes with resistance in a range of *S. frugiperda* strains can now be examined, such studies combined with heterologous expression and functional analysis of putative resistance proteins will identify which actually confer resistance.

Overall the information provided by this study is a prerequisite for the design, implementation and monitoring of resistance management strategies for *S. frugiperda* that aim to preserve the efficacy of the insecticide classes used for control. Given that there is a limited arsenal of effective chemical classes for the control of *S. frugiperda* and the current reliance on the use of organophosphates and pyrethroids in Brazil (see introduction) means such strategies are urgently required.

## Supporting Information

Table S1 **Oligonucleotide primers used in this study.**
(XLS)

Table S2 **ESTs significantly differentially transcribed (more than 2-fold over or under expressed, p<0.01) between the OP strain and the susceptible SUS strain in microarray analysis.**
(XLS)

Table S3 **ESTs significantly differentially transcribed (more than 2-fold over or under expressed, p<0.01) between the PYR strain and the susceptible SUS strain in microarray analysis.**
(XLS)
